# Author Correction: Improving the diagnosis of thyroid cancer by machine learning and clinical data

**DOI:** 10.1038/s41598-022-17659-1

**Published:** 2022-08-02

**Authors:** Nan Miles Xi, Lin Wang, Chuanjia Yang

**Affiliations:** 1 Department of Mathematics and Statistics, Loyola University Chicago, Chicago, IL 60660 USA; 2 Department of Statistics, Purdue University, West Lafayette, IN 47907 USA; 3 Department of General Surgery, Shengjing Hospital of China Medical University, Shenyang, 110004 Liaoning China

Correction to: *Scientific Reports* 10.1038/s41598-022-15342-z, published online 01 July 2022

The Funding section in the original version of this Article was omitted. The Funding section now reads:

“The present study was supported by the 345 Talent Project of Shengjing Hospital (grant no. M0731).”

The original Article has been corrected.

